# Model of inverse bleb growth explains giant vacuole dynamics during cell mechanoadaptation

**DOI:** 10.1093/pnasnexus/pgac304

**Published:** 2022-12-23

**Authors:** Andrea Cairoli, Alice Spenlehauer, Darryl R Overby, Chiu Fan Lee

**Affiliations:** Department of Bioengineering, Imperial College London, London SW7 2AZ, UK; Department of Bioengineering, Imperial College London, London SW7 2AZ, UK; Department of Bioengineering, Imperial College London, London SW7 2AZ, UK; Department of Bioengineering, Imperial College London, London SW7 2AZ, UK

**Keywords:** shape mechanoadaptation, coarse-grained modeling, inverse blebbing, Ostwald ripening

## Abstract

Cells can withstand hostile environmental conditions manifest as large mechanical forces such as pressure gradients and/or shear stresses by dynamically changing their shape. Such conditions are realized in the Schlemm’s canal of the eye where endothelial cells that cover the inner vessel wall are subjected to the hydrodynamic pressure gradients exerted by the aqueous humor outflow. These cells form fluid-filled dynamic outpouchings of their basal membrane called *giant vacuoles*. The inverses of giant vacuoles are reminiscent of cellular blebs, extracellular cytoplasmic protrusions triggered by local temporary disruption of the contractile actomyosin cortex. Inverse blebbing has also been first observed experimentally during sprouting angiogenesis, but its underlying physical mechanisms are poorly understood. Here, we hypothesize that giant vacuole formation can be described as inverse blebbing and formulate a biophysical model of this process. Our model elucidates how cell membrane mechanical properties affect the morphology and dynamics of giant vacuoles and predicts coarsening akin to Ostwald ripening between multiple invaginating vacuoles. Our results are in qualitative agreement with observations from the formation of giant vacuoles during perfusion experiments. Our model not only elucidates the biophysical mechanisms driving inverse blebbing and giant vacuole dynamics, but also identifies universal features of the cellular response to pressure loads that are relevant to many experimental contexts.

Significance Statement Human Schlemm’s canal endothelial cells in physiological conditions are subjected to a pressure gradient caused by the flow of aqueous humor in the basal-to-apical direction across the endothelium leading to the formation of cellular outpouchings called giant vacuoles. The physical mechanisms regulating giant vacuole formation are unknown. By describing giant vacuoles as inward blebs, we formulate a model of their growth and collapse that captures the characteristic features observed experimentally. Our theory reveals that the abrupt increase in surface tension caused by membrane stretching, which is required to accommodate the large areal strains locally induced by inward blebbing, limits giant vacuole growth. The model also predicts a competition between multiple invaginating vacuoles, in which big vacuoles win over small vacuoles.

## Introduction

Morphological regulation is crucial for the survival of eukaryotic cells in physiological conditions (1). Shape changes are involved in a broad spectrum of cellular functions, such as motility and division (2, 3), and represent the main cellular response to large mechanical or osmotic pressure gradients and shear stresses (4). Exemplary forces of this type are found especially in the Schlemm’s canal of the human eye. Schlemm’s canal belongs to the conventional outflow pathway of aqueous humor (5), a transparent fluid that bathes the lens and other tissues of the eye and is the major determinant of intraocular pressure. As elevated intraocular pressure is a risk factor for glaucoma (6), its regulation is a key physiological process, primarily realized by draining aqueous humor through the inner wall endothelium of Schlemm’s canal (7). This is a continuous monolayer of spindle-shaped endothelial cells, that are attached between one another by tight junctions and adhere to a discontinuous basement membrane (8). These cells are subjected to the hydrodynamic pressure exerted by the aqueous humor outflow [typically about 1 mmHg in healthy human eyes (9)], which corresponds to a significant force (in cellular terms) in the basal-to-apical direction [about 60 nN (10)].

Under these conditions, Schlemm’s canal endothelial cells form large dome-like fluid-filled outpouchings, called *giant vacuoles* (GVs) (11–13), by locally invaginating the basal aspect of the cell surface into the cellular body. These structures are typically oriented along the cell axis and are entirely bounded by a smooth unit membrane, i.e., their inner cavity remains extracellular at all times. They thus exhibit a “signet ring” shape: The cell appears as a thin, continuous lining around the GV cavity with the nucleus bulging to one side. Interestingly, these structures are not

unique to Schlemm’s canal endothelial cells but can also be found in other cell types (19–22).

GVs have been studied experimentally both in vivo and in vitro [see reviews (10, 23)]. All these experiments revealed several characteristic features of GVs, here briefly summarized. (i) The formation of GVs depends on the pressure drop $\Delta p$ across the inner wall endothelium that drives the growth process of GVs (24–27). When a positive $\Delta p$ is established, GVs form within circa 15 min (28); upon removal of the pressure drop, they shrink within a timescale of the order of minutes (29, 30) (survival time). (ii) GVs typically induce large increase of the net surface area of Schlemm’s canal cells [by three-fold to four-fold per vacuole when the intraocular pressure is increased from 15 to 30 mmHg according to the data in ref. (27); see details in ref. (10)]. Finally, (iii) their morphology depends on the cell surface tension: reduction of the cortical tension through impairment of cellular actomyosin contractility leads to larger GVs (31).

Despite the abundance of experimental studies, however, the biophysical mechanisms underlying GV formation have not yet been fully understood. To accommodate the large shape deformations associated with GVs, cells regulate their surface area through a complex dynamical process, which involves remodeling of the cell membrane and reorganization of the actomyosin cortex, orchestrated by the surface tension (32–37). This process promotes the formation of many different cellular protrusions, such as blebs (15, 38, 39), microvescicles (40), and either tubular or spherical membrane invaginations (reminiscent of GVs) (32, 41, 42). For the underlying dynamical picture, a natural timescale of the order of minutes has been reported (43): At shorter times, the protrusions are rapidly formed via a local passive mechanical process; on longer times they are reabsorbed via active actin- and temperature-dependent contractile processes.

In the context of angiogenesis, a similar process also occurs and has been termed *inverse blebbing* (44). Specifically, the authors there proposed a qualitative picture, in which the hydrodynamic pressure gradient exerted by the blood flow on the apical aspect of an endothelial cell, promoted by local weakening of the cell actomyosin cortex, drives the nucleation of a spherical membrane invagination within the cell body. This protrusion is then mechanically inflated, while simultaneously F-actin polymerization is initiated on site and myosins-II motors are recruited from the cytoplasm to build a contractile actin cortex that fully envelopes and potentially stabilizes the invaginated structure. This mechanism not only is reminiscent of cell blebbing with an inverted polarity [ordinary blebs are extracellular spherical protrusions of the cytoplasm (38)], but also bears similarities with the formation of GVs in Schlemm’s canal endothelial cells (which are, however, typically ellipsoidal).

Inspired by these analogies, here, we formulate a quantitative model of GV formation that can reproduce all the qualitative features (i) to (iii). Our model shows that cell membrane is central to the mechanics of GV formation, in particular by enabling cells to limit the growth of the vacuoles, thus confirming previous similar conjectures (10). The model also predicts coarsening effects between multiple invaginating GVs, akin to Ostwald ripening in phase separation (45–48), which are induced by their local competition for membrane stretching. The characteristic features (i) to (iii) and our own model predictions (see the “Discussion” section) can be relevant also in other experimental contexts where cells respond to large pressure gradients through inverse blebbing (49).

## Single-Cell Perfusion Model

### Initial setup and cell mechanical model

**Fig. 1.**
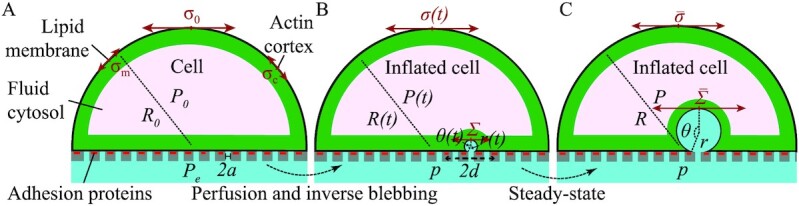
Schematic of the cell mechanical model. (A) Initial configuration. A cell adheres to a filter that is immersed in a fluid at the pressure ${{P}}_{e}$. The cell is composed by the plasma membrane with tension ${{{\sigma }}}_{\rm{m}}$, the actomyosin cortex with tension ${{{\sigma }}}_{\rm{c}}$, and the cytosolic fluid at the pressure ${{{P}}}_0 \ge {{{P}}}_{{e}}$. The cell surface tension (membrane plus cortex) is ${{{\sigma }}}_0$. (B) Time-dependent inverse bleb configuration. The inverse bleb is a spherical cap with radius ${{r}}$ and opening angle ${{\theta }}$. Its overall surface tension is ${{\Sigma }}$. Inverse bleb tension equilibrates over a membrane patch of radius ${{d}}$. The cell maintains hemispherical shape with radius ${{R}}$, internal pressure ${{P}}$, and surface tension ${{\sigma }}$. (C) Steady-state configuration after perfusion at constant pressure drop. For sufficiently long times (up to a few tens of minutes; comparable with the typical lifetime of GVs), the inverse bleb is enveloped by a contractile actomyosin cortex. The surface tensions of the cell and the inverse bleb, denoted as ${{\bar{\sigma }}}$ and ${{ {\bar {\Sigma }}}}$, respectively, are equilibrated locally.

We recapitulate the notation defined in this Section in SI Appendix, Table S1. We report physiological values of all model parameters in Table 1. We consider an endothelial cell that adheres to a filter immersed in a fluid environment at the controlled pressure ${P}_e$ (Fig. 1A). At first approximation, the cell at equilibrium assumes a hemispherical shape with radius ${R}_0$ due to its effective surface tension (2). The filter pores have width $2a$, which is consistent with the characteristic diameter of the meshwork pores of GVs (11, 14).

**Table 1.** Summary of the model parameters.

**Parameter**	**Value**	**Units**	**Reference**
${R}_0$	$9 - 11$ ^a^	$\mu {\rm{m}}$	(8)
$2a$	$0.5$ ^b^	$\mu {\rm{m}}$	(14)
${\sigma }_{\rm{m}}$	$40$	${\rm{pN/}}\mu {\rm{m}}$	(15)
${\sigma }_{\rm{c}}$	$374$	${\rm{pN/}}\mu {\rm{m}}$	(15)
${K}_{\rm{m}}$	${10}^5$	${\rm{pN/\mu m}}$	(2)
*d*	$5 - 12$ ^c^	$\mu {\rm{m}}$	(16)
${\varepsilon }^{\rm{*}}$	$0\% - 100{\rm{\% }}$	${\rm{adim}}$	(17)
${\tau }_{\rm{c}}$	$1$	${\rm{s}}$	(18)
$\mu $	$2.5 \times {10}^4$	${\rm{pN}} \times {\rm{s/}}\mu {{\rm{m}}}^2$	

a Human Schlemm’s canal cells are spindle-shaped with length, width, and height, respectively, 100, 4 to 8, and 5 μm (8). Assuming elliptic shape, their surface area is 900 to 1110 μm^2^. Setting it equal to $3\pi R_0^2$ yields the given estimate for *R*_0_.

b The estimate reported was measured in ref. (14) at physiological intraocular pressure, 15 mmHg. This corresponds to a pressure drop about 7 mmHg. In general, the meshwork pore width is shown to be dependent on the imposed pressure drop.

c Membrane tension was shown in ref. (16) to propagate diffusively with diffusion coefficient $D\ = \ 0.024\mu {{\rm{m}}}^2{\rm{/s}}$. The characteristic lifetime of GVs is of the order of a few tens of minutes, say $t \approx 10 - 50\ {\rm{min}}$. The estimate for the radius of the membrane patch where tension equilibrates is given by$\sqrt {2Dt} $.

To describe the cell, we consider the following mechanical model (Fig. 1A): The cell is coated by a lipid membrane with in-plane surface tension ${\sigma }_{\rm{m}}$ and area expansion modulus ${K}_{\rm{m}}$ (2). The latter is a mechanical parameter describing the resistance exerted by the membrane to elastic deformations of its area upon application of a stress. While the former is due to purely entropic effects, the latter determines the elastic response of the membrane to mechanical stretch and is much larger in value (Table 1). Lying beneath the membrane and anchored to it, the cell possesses a contractile actin cortex, a dense cross-linked meshwork of actin filaments, myosin motors, and actin-binding proteins (50, 18). An in-plane tension ${\sigma }_{\rm{c}}$ is generated in the cortical network primarily by myosin-II motors (51, 52). Prior to perfusion, the membrane is floppy and only the entropic surface tension contributes. We thus set the overall cell surface tension to be ${\sigma }_0 = {\sigma }_{\rm{m}}\ + {\sigma }_{\rm{c}}$ (51). The cell interior is filled with cytosolic fluid at the pressure ${P}_0\geqslant{P}_e$. Differently from previous models of ordinary cell blebbing (15), other internal elastic contributions, such as cytoskeletal components and cellular organelles, are neglected. In the present context, these only exert resistance to inverse bleb inflation, an effect that we will capture at a coarse-grained level (see below). The equilibrium configuration of the cell is determined by the Laplace law ${P}_0 - \ {P}_e = \ 2{\sigma }_0/{R}_0$.

### Inverse blebbing and perfused configuration

**Fig. 2.**
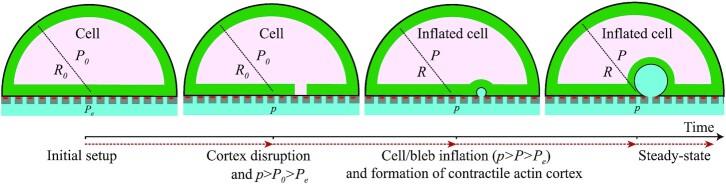
Schematic of inverse blebbing. See ref. (44) for details.

We can now perfuse the model cell by increasing the pressure of the ambient fluid inside the filter to $p \ge {P}_e$. We consider only constant pressure, but any time-dependent protocol $p( t )$ can be studied using the same method. This condition leads to the formation of an invagination of the cell basal aspect within the cellular body through inverse blebbing (44) (Fig. 2): The hydrodynamic pressure drop $p - {P}_0$ across the cell basal surface together with a local disruption of the actomyosin cortex drives the nucleation of an intracellular membrane protrusion^a^. Both this inverse bleb and the cell then start inflating. Here, we make the simplifying assumption that the cell inflates while keeping fixed its hemispherical shape. This implies that the cell adds new focal complexes instantaneously to cover its expanding basal surface [this is compatible with previous observations on spreading cells, see ref. (53)]. Simultaneously, actin polymerization and myosin-II motors recruitment are initiated on the inverse bleb surface, such that a contractile actin shell, akin to the cell cortex, is formed around the inverse bleb. With time, the shell grows to the same thickness of the cell cortex, such that its surface tension becomes equal to the cortical tension. For sufficiently long times, the system can reach mechanical equilibrium, where the surface tensions of both the contractile shell and the plasma membrane balance the pressure force exerted at the cell–bleb interface. This mechanism is reminiscent of ordinary cellular blebbing (38). The crucial difference is that inverse blebs have opposite polarity, in that they inflate inside the cellular body. In addition, while blebs are self-sustained through the cytoplasmic pressure, inverse blebs require an external pressure drop.

We assume that the inverse bleb has spherical cap shape with radius *r* and opening angle $\theta $ ($\pi /2 \le \theta \le \pi $)^b^, which are related by the geometrical condition $r{\rm{sin\ }}( \theta ) = \ a$. The instantaneous surface tension of the inverse bleb is denoted by $\Sigma $. Likewise, the cell maintains its initial hemispherical shape but with radius *R*, internal pressure *P*, and surface tension $\sigma $ (Fig. 1B). We remark that at nucleation $\Sigma \ = {\sigma }_{\rm{m}}\ $, because cortical disruption (see above) causes a local reduction and/or impairment of the surface tension in the region of the cell basal aspect with radius *a*, where the inverse bleb resides.

### Modeling inverse bleb growth

To enable the inflation of the inverse bleb within the cellular body, the pressure inside the inverse bleb *p* must be larger than the intracellular pressure *P* (54). The membrane mechanical behavior is typically described in terms of the fluid-mosaic model (55). This model prescribes that the membrane can be regarded effectively as a 2D viscous fluid. This implies that the membrane tensions of the cell and the inverse bleb should be able to equilibrate globally even at short times (e.g., a few seconds). Considering that actin cortical dynamic is also fast (see Table 1) for both the cell and the inverse bleb, their surface tensions all together (membrane plus cortex contribution) should also be able to equilibrate globally even at short times. This expectation, however, has been refuted by recent experiments of membrane tethering. These experiments have demonstrated that the surface tension of the inverse bleb and the cell can only equilibrate locally for short to intermediate timescales (up to about 10 min) (16). This counterintuitive effect is caused by the impediment to tensional flow exerted by cytoskeleton-bound transmembrane proteins^c^. As a result, for timescales up to a few tens of minutes, the surface tensions of the cell and of the inverse bleb can be different. Over these timescales, the inverse bleb tension equilibrates within a membrane patch of radius $d \simeq 10\ \mu {\rm{m}}$ (see Table 1). Therefore, *d* is a parameter that describes the radius of the region in the cell basal aspect wherein the inverse bleb surface tension can effectively equilibrate for times up to the typical lifetime of GVs. For longer timescales, the inverse bleb and the cell interact through the slow lipid flow that is driven by the tensional gradient established at their boundary (16). This enables equilibration of surface tension throughout the entire system at long times. However, because the characteristic lifetime of GVs is of the order of a few tens of minutes, we ignore the eventual equilibration and consider their tension separately (Fig. 1C).

### Inverse bleb radius dynamic: the Rayleigh–Plesset equation

Given the above discussion, we now formulate a dynamical model for GVs. The key ingredients are (1) the inverse bleb growth dynamic is overdamped^d^ and is driven by the mechanical pressure, viscous resistance, and surface tension forces that are exerted uniformly at the cell–bleb interface. (2) The intracellular pressure *P* is fast equilibrating, i.e., its rate of change is negligible compared to the corresponding rates of the tensions and the radii. (3) Finally, there is no leakage of intracellular material to the extracellular environment during inverse blebbing. This condition is experimentally supported (19) and prescribes volume conservation at all times. Consequently, the cell radius can be specified by the geometrical relation ${R}^3 = R_0^3\ + 2{r}^3( {2 + \cos \theta } )\sin {( {\theta /2} )}^4$. The volumetric contribution of the basal pore can be neglected (SI Appendix Section I).

Taken all altogether, the above assumptions led us to model the dynamic of the radius of the inverse bleb in terms of the Rayleigh–Plesset equation without the inertial terms (58–60):

(1) \begin{eqnarray*} \frac{{{\rm{d}}r\left( t \right)}}{{{\rm{d}}t}} = \frac{{r\left( t \right)}}{{4\mu }}\ \left[ {p - P\left( t \right) - \frac{{2\Sigma \left( t \right)}}{{r\left( t \right)}}} \right], \end{eqnarray*}

where $\mu $ describes the resistance to inverse bleb growth exerted by the inner cell body. We can interpret the parameter $\mu $ as an effective dynamic viscosity that captures at a coarse-grained level both the viscous drag force exerted by the cytoplasm on the growing inverse bleb and the resistive forces exerted by other components of the cell inner body. This equation expresses balance of the mechanical force due to the bleb surface tension, and of the mechanical pressure and viscous forces exerted by the inner cell body at the cell–bleb interface. The intracellular pressure is set by the mechanical equilibrium prescribed by the Laplace law for the cell $P( t ) - \ {P}_e = 2\sigma ( t )/R( t )\ $ (Fig. 1B). The surface tensions ${\rm{\Sigma }}$ and $\sigma $ are functions of the relative surface area strains of the inverse bleb and cell, respectively (see below). As such, they are functions of the respective radii, *r* and *R*. In turn, these specify their time dependence. Complemented by volume conservation, which relates the variables *R* and *r*, and by the geometrical relation between $\theta $ and *r*, these equations only depend on the independent variables $( {r,\Sigma ,\sigma } )$ and can be solved numerically for fixed pressure drop $\Delta p \equiv p - {P}_e$.

In particular, the steady-state solutions of these equations (Fig. 1C) are determined by

(2) \begin{eqnarray*} \Delta p\ = \frac{{2{\rm{\bar{\Sigma }}}\left( r \right)}}{r}\ + \frac{{2\bar{\sigma }\left( r \right)}}{R}. \end{eqnarray*}

We stress that this limit describes the system configurations for timescales close to the characteristic lifetime of GVs, but small enough that the lipid flow established at the inverse bleb–cell interface can be neglected. The equation is closed and can be solved for *r* in terms of $\Delta p$. We note also that correction terms due to membrane bending rigidity are expected to be negligible (SI Appendix Section II).

### Surface tension dynamic: dependence on the relative area strain

**Fig. 3.**
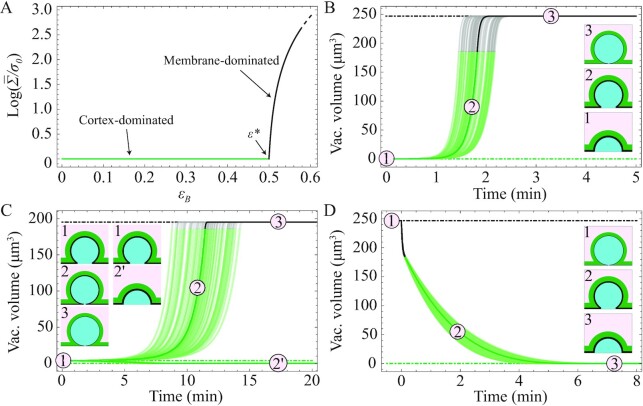
Dynamics of inverse blebs. Model parameters are chosen according to Table 1, in particular ${R}_0 = \ 10\ \mu {\rm{m}}$, $d\ = \ 10\ \mu {\rm{m}}$, and ${\varepsilon }^* = \ 0.5$. The pressure drop is kept constant for all times. Blurred regions correspond to trajectories obtained by allowing for up to $20{\rm{\% }}$ variability in the dynamical parameters ${\tau }_{\rm{c}}$ and $\mu $. We plot simulation results for 100 different sets of parameters chosen randomly. We also plot schematics of inverse bleb configurations to illustrate the temporal evolution of the geometrical shape. (A) Steady-state surface tension of the vacuole vs. its surface area strain. Cortex- and membrane-dominated mechanical regimes are highlighted with different colors (green/black, respectively). We assume the same functional form for the cell. (B) Large pressure drops. We set here $\Delta p\ = \ 30\ {\rm{mmHg}}$. The giant vacuole is initially nucleated with $r\ = \ a$ and $\Sigma \ = {\sigma }_{\rm{m}}\ $. At this pressure, the inverse bleb inflates until it reaches the steady-state giant vacuole configuration in the membrane-dominated regime (within about $2\ {\rm{min}}$). Then growth stops. (C) Physiological pressure drops. We set here $\Delta p\ = \ 7\ {\rm{mmHg}}$. The dynamic become sensitive on the nucleation conditions. Different nucleation radii can lead to either inverse bleb shrinkage or growth. (D) Giant vacuole collapse. The giant vacuole is initially set in its steady-state configuration (at $\Delta p\ = \ 30\ {\rm{mmHg}}$). At time $t\ = {0}^ + \ $, the pressure drop is removed. The giant vacuole slowly deflates (within about $5\ {\rm{min}}$).

The surface tensions of both the inverse bleb and the cell (considered separately; see above) follow a relaxation dynamic toward their value at steady state, ${\rm{\bar{\Sigma }}}$ and $\bar{\sigma }$, respectively. In our context, both this target tension and the characteristic timescale of relaxation are determined dynamically during the growth process through a dependence on the relative area strains, ${\varepsilon }_{\rm{B}}$ for the bleb and ${\varepsilon }_{\rm{C}}$ for the cell. We define ${\varepsilon }_{\rm{C}}( t ) \equiv [ {A( t ) - {A}_0} ]/{A}_0$ with ${A}_0 = \ 3\pi R_0^2 - \pi {d}^2$ and $A\ = \ 3\pi {R}^2 - \pi {d}^2$; ${\varepsilon }_{\rm{B}}( t ) \equiv [ {S( t ) - {S}_0} ]/{S}_0$ with ${S}_0 \equiv \pi {d}^2$ and $S\ = \ 2\pi {r}^2( {1 - \cos \theta } ) + \pi ( {{d}^2 - {a}^2} )$. We assume two mechanical regimes as a function of these relative area strains (Fig. 3A).

#### Cortex-dominated regime

The plasma membrane can adapt to possibly large relative area strains at constant surface tension [up to ${\varepsilon }^{\rm{*}} = \ $ 100% at cellular level^e^; see ref. (17)]. Different pathways have been identified that enable cells to buffer the surface area required (62). The membrane possesses different types of lipid reservoirs, e.g., caveolae (63, 64), membrane wrinkles and invaginations (65, 66), microvilli and other membrane protrusions (67, 68), that can be flattened out. Additional membrane can also be supplied by active exocytic processes within timescales of seconds to minutes (43), thus comparable with the typical lifetime of GVs (see above). The dominant contribution to the steady-state tension in this regime is provided by the actomyosin cortex for the cell and by the actin contractile shell for the inverse bleb. As such, ${\rm{\bar{\Sigma }}} = {\sigma }_0\ $ and ${\rm{\bar{\sigma }}} = {\sigma }_0\ $. The cell cortex is already initialized at its steady-state value. Therefore, if the cell remains in this regime, its surface tension does not change in time, i.e., $\sigma ( t ) = {\rm{\bar{\sigma }}} = {\sigma }_0 $. The contractile shell enveloping the inverse bleb must be rebuilt instead following the local disruption of the cortex occurring at nucleation. Correspondingly, tensional dynamic is governed by the characteristic timescale ${\tau }_{\rm{c}}$ of actin turnover and myosin recruitment (18). Assuming exponential relaxation, we can write

(3) \begin{eqnarray*} \frac{{{\rm{d}}\Sigma \left( t \right)}}{{{\rm{d}}t}} = \frac{1}{{{\tau }_{\rm{c}}}}\ \left[ {{\sigma }_0 - \Sigma \left( t \right)} \right]. \end{eqnarray*}

We remark that this equation can be interpreted as a Maxwell model describing the stress-strain relation of a viscoelastic material with a strain rate proportion to ${\sigma }_0$. This means that we are treating the actin cortex as a viscoelastic material with an imposed strain rate prescribed by the mechanical equilibrium at the cell–inverse bleb interface.

#### Membrane-dominated regime

Once the membrane reservoirs are depleted, which corresponds to the threshold relative area strain ${\varepsilon }^{\rm{*}}$, no additional lipids can be added to the plasma membrane, such that further increase in the surface area must be obtained by mechanically stretching the membrane. This mechanical process induces abrupt increase of the surface tension due to the large area expansion modulus of the membrane (2). Therefore, the dominant contribution to the surface tension in this regime is given by the taut membrane. Assuming exponential relaxation, we can write

(4) \begin{eqnarray*} \frac{{{\rm{d}}\Sigma \left( t \right)}}{{{\rm{d}}t}} = \frac{1}{{{\tau }_{\rm{m}}}}\ \left[ {{\rm{\bar{\Sigma }}}\left( t \right) - \Sigma \left( t \right)} \right], \end{eqnarray*}

where we denote ${\tau }_{\rm{m}}$ the characteristic timescale of tension equilibration in the taut membrane and the equilibrated tension ${\rm{\bar{\Sigma }}}\ ( t ) = {\sigma }_0 + {K}_{\rm{m}}[ {{e}^{( {( {S( t ) - {S}^*} )/{S}^*} )} - 1} ]$, with ${S}^* = {S}_0\ ( {1 + {\varepsilon }^*} )$. This is a phenomenological model based on experimental observations (69)^f^. However, in these conditions, the membrane is in the 2D liquid-like state (55), such that its equilibration is a fast process compared to all other relevant cellular processes (including actin turnover). For all intents and purposes, the equilibration process in this regime can be considered as instantaneous. Therefore, taking the limit ${\tau }_{\rm{m}} \to 0$ in Eq. (4), we obtain ${\rm{\Sigma }}( t ) = {\rm{\bar{\Sigma }}} ( t )$. In our context, this regime is only relevant for the inverse bleb. In fact, when only one GV is formed, the area strain of the cell never reaches the threshold ${\varepsilon }^*$ (up to at least 30 mmHg).

## Results

### Inverse blebs dynamics

Numerical analysis of the model equations enables us to investigate the dynamical features of inverse blebbing. We adopt the following protocol: The pressure drop $\Delta p$ is established at the initial time $t\ = \ 0$ and kept constant throughout the growth process. The inverse bleb is initially invaginated with $\Sigma \ = {\sigma }_{\rm{m}}\ $. This condition replicates the local disruption of the actomyosin cortex observed during inverse bleb nucleation (44). We set reference values for all model parameters according to Table 1 (for exact values, see caption of Fig. 3).

We observe three distinct qualitative scenarios (Fig. 3): For sufficiently small ${\rm{\Delta }}p$, the inverse bleb immediately deflates independently of its radius at nucleation (not shown). By construction, our model can only predict the deflation dynamic of the inverse bleb until $r\ = \ a$. This result suggests the existence of a threshold pressure for GV formation, $\Delta {p}^{\rm{*}}$. For large (and rather nonphysiological) pressure drops the inverse bleb grows toward a target configuration within a characteristic timescale of the order of minutes. Then, growth stops (Fig. 3B). This target configuration corresponds to the steady-state GV described by Eq. (2). The growth process in this case is again independent of the inverse bleb radius at nucleation. For intermediate pressure drops, the dynamic of the inverse bleb becomes sensitive on the nucleation radius. For sufficiently small radii, inverse blebs shrink; conversely, for large enough radii, inverse blebs grow (Fig. 3C). This result suggests the existence of a threshold nucleation radius for GV growth at fixed physiological pressure drops.

We can also study GV collapse (Fig. 3D). The GV is initialized at its steady-state configuration for pressures $\Delta p > \Delta {p}^\dagger $ (here, $\Delta p\ = \ 30\ {\rm{mmHg}}$). At time $t\ = {0}^ + \ $, the pressure drop is removed and the GV let evolve according to Eqs. (1), (3), and (4). The model predicts shrinkage of the GV within a larger timescale than the corresponding timescale for growth at the same pressure (here about $4 - 6\ {\rm{min}}$). Moreover, we observe an even longer timescales for GV collapse if the cortical tension is reduced (SI Appendix Section III; Fig. S1).

We study how robust these results are against physiological variability of the dynamical parameters, ${\tau }_{\rm{c}}$ and $\mu $, by solving the model while allowing for up to 20% change in their reference values. The qualitative results discussed above are confirmed in all cases.

### Inverse blebs steady-state configurations

**Fig. 4.**
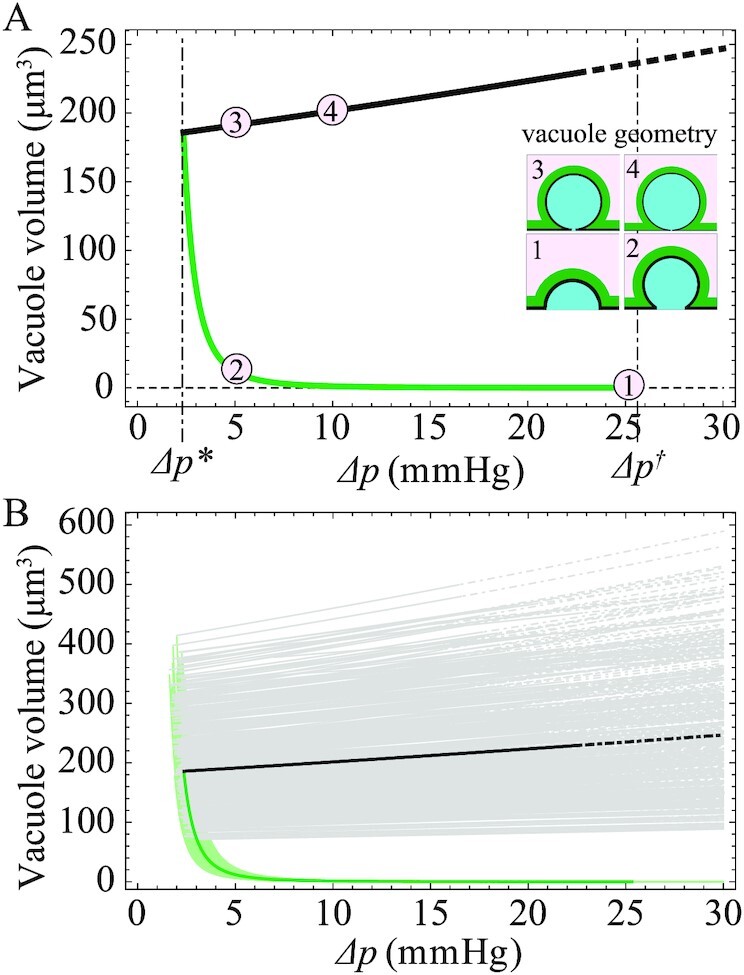
Steady-state configurations of GVs. (A) Vacuole volume vs. perfusion pressure drop $\Delta p$. Schematics of vacuole configurations are plotted to illustrate the dependence of the geometrical shape on the pressure drop. Characteristic parameters in panels A and B are chosen according to Table 1. For $\Delta p$ large, the surface tension can reach lytic values (here corresponding to area strains 5% ${\varepsilon }^{\rm{*}}$), when rupture of the plasma membrane can occur (dot-dashed line). (B) Steady-state configurations obtained by allowing for up to 20% variability in the characteristic parameters of the model. We plot numerical solutions for 500 different sets of parameters chosen randomly. These results indicate that single-cell predictions are qualitatively robust against physiological tissue variability.

Numerical analysis of the steady-state Eq. (2) confirms the previous results (Fig. 4A). For large enough pressures ($\Delta p \ge \Delta {p}^\dagger $), only one steady-state configuration exists. This explains why inverse blebs grow to this GV configuration independently of the nucleation radii at these pressures. For intermediate pressures ($\Delta {p}^{\rm{*}} \le \Delta p \le \Delta {p}^\dagger $), two steady-state configurations exist with ${\rm{\bar{\Sigma }}}$ either in the cortex- or in the membrane-dominated regime. Using linear stability analysis, we can study how steady-state inverse blebs respond to small perturbations of their shape. We find that inverse blebs in the membrane-dominated regime are stable, while those in the cortex-dominated regime are unstable (SI Appendix Section IV; Fig. S2). The dynamical picture becomes clear: If the inverse bleb is nucleated with a radius smaller than the radius of the steady-state configuration in the cortex-dominated regime, it will shrink. Conversely, if it is nucleated with a larger radius, it will grow toward the steady-state GV configuration in the membrane-dominated regime. The threshold nucleation radius for GV growth at fixed intermediate pressures is therefore of the order of the radius of the steady-state configuration in the cortex-dominated regime. We remark that cells can ensure stability of the GVs in the cortex-dominated regime by enveloping them in additional contractile actomyosin structures (like actin rings) that respond elastically (at short times) to the perturbation (15) (SI Appendix Section IV).

These considerations also yield an estimate of the GV nucleation pressure, $\Delta {p}^{\rm{*}} \simeq 2{\sigma }_0( {R + r} )/Rr$, where *R* and *r* are both ${\varepsilon }^{\rm{*}}$-dependent^g^. This function decreases monotonically as *r* increases (due to volume conservation) independently of the specific geometry of the perfused system. As such, $\Delta {p}^{\rm{*}}$ only depends on the membrane reservoir size through the parameter ${\varepsilon }^{\rm{*}}$ and on the initial cell surface tension ${\sigma }_0$ (linearly).

All these results are qualitatively robust against physiological tissue variability. This is confirmed by numerically solving Eq. (2) while allowing for up to 20% change in the model parameters (chosen randomly). All stationary configurations obtained do not differ qualitatively between themselves and from the reference case (Fig. 4B). We remark that the robustness of our model results against variability of the parameter $2a$ (the pore diameter) is especially relevant, because in physiological tissues, Schlemm’s canal endothelial cells adhere to a discontinuous basement membrane possessing a broad distribution of pore sizes (SI Appendix Section V).

### Coarsening of multiple giant vacuoles through Ostwald ripening

In physiological conditions, multiple GVs typically invaginate simultaneously. As a proxy of this general situation, we study the case of two growing GVs. Neglecting steric interactions between the blebs- and contact-driven shape deformations, Eqs. (1), (3), and (4) are formally valid for both the cell and each inverse bleb. However, the volume conservation must now account for the contribution of all blebs; therefore, the relation determining the cell radius becomes ${R}^3 = R_0^3\ + 2\mathop \sum \limits_i r_i^3( {2 + {\rm{cos}}{\theta }_i} ){\rm{si}}{{\rm{n}}}^4( {{\theta }_i/2} )$ ($i\ = \ 1,2$).

**Fig. 5.**
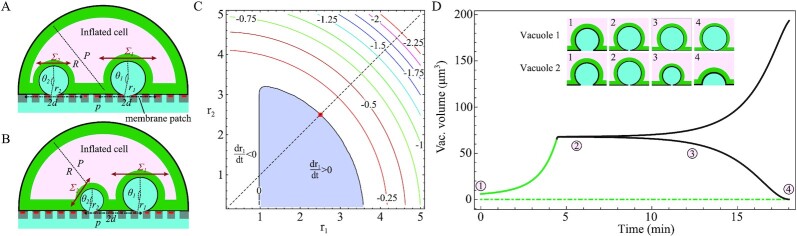
Coarsening process between multiple invaginating GVs. (A) Two vacuoles invaginate simultaneously at a distance larger than *d*. Because they do not share the same equilibrating membrane patch, they grow independently. (B) Two vacuoles invaginate simultaneously at a distance smaller than *d*. The vacuoles share the same equilibrating membrane patch. Their growth processes are no longer independent. (C) Contour plot of $d{r}_1/dt$ vs. $({r}_1,{r}_2)$ at steady state. The pressure drop is set at physiological values, here $\Delta p\ = \ 7\ {\rm{mmHg}}$. Other model parameters are set according to Table 1, in particular ${R}_0 = \ 10\ \mu {\rm{m}}$, $d\ = \ 10\ \mu {\rm{m}}$, and ${\varepsilon }^* = \ 0.5$. The structure of contours is similar to the standard drop coarsening picture as in the Ostwald-ripening scheme. (D) Time series of the volume of two GVs illustrating the coarsening dynamic. Model parameters are chosen as in panel C. Schematics illustrate the temporal evolution of the shape of the GVs. The two GVs are initialized simultaneously at time $t\ = \ 0$ with different, randomly chosen, initial sizes close to their steady-state configuration in the cortex-dominated regime, here ${r}_1 \approx 1.1238$ and ${r}_2 \approx 1.1241$, and ${{\rm{\Sigma }}}_i = {\sigma }_{\rm{m}}\ $.

We can now distinguish two different qualitative scenarios: First, the GVs can be nucleated at a distance larger than the characteristic length-scale *d* (see above), such that they grow by increasing the surface area of two different membrane patches (Fig. 5A). These vacuoles are therefore fully independent, and their steady-state and dynamical properties are the same as those of a single GV. Second, the GVs can be nucleated at a distance smaller than *d*, meaning that they grow by sharing the same membrane patch (Fig. 5B). The two vacuoles are therefore no longer independent: Their surface tensions converge to the same target tension $\bar{\Sigma }$ dependent on the relative area strain ${\varepsilon }_B$ with $S\ = \sum \limits_i r_i^2\ ( {2 + \cos {\theta }_i} ){\rm{si}}{{\rm{n}}}^4( {{\theta }_i/2} ) + \pi ( {{d}^2 - 2{a}^2} )$. The steady-state configurations predicted by the model do not change qualitatively from the single-inverse bleb picture: The second bleb only reduces the amount of stored membrane locally available for each inverse bleb. Consequently, the threshold pressure for GV nucleation $\Delta {p}^{\rm{*}}$ increases and the maximal size of each vacuole decreases.

When we consider the dynamics of the vacuoles, we observe a coarsening mechanism akin to Ostwald ripening. This is demonstrated by the contour plot of $d{r}_1/{\rm{d}}t$ as a function of ${r}_1$ and ${r}_2$ (Fig. 5C) at steady state (i.e., ${{\rm{\Sigma }}}_1 = {{\rm{\Sigma }}}_2\ = {\rm{\bar{\Sigma }}}\ $ and $\sigma \ = {\rm{\bar{\sigma }}}\ $). The structure of the contours is similar to the standard drop coarsening picture as in the Ostwald-ripening scheme. Focusing on the equal size configuration (${r}_1 = {r}_2\ $) at the zero-contour line (indicated by a red square), fluctuations (e.g., increasing ${r}_1$ while decreasing ${r}_2$) around that point can generate an instability that leads to the growth of the bigger GV and the shrinking of the smaller one.

The dynamic of the coarsening process can also be characterized (Fig. 5D): Consider two GVs, nucleated with different sizes, that grow simultaneously. Two scenarios can occur: If the two vacuoles remain small enough that the cell can fully buffer the area increase required through its local (i.e., within the patch of radius *d*) membrane reservoirs, the two vacuoles grow independently of one another. Conversely, if the two vacuoles become large enough to exhaust the membrane reservoirs, they enter the membrane-dominated regime and start competing between one another in order to stretch locally the lipid membrane and increase their own surface area. For long times, symmetry breaking occurs: Only one GV succeeds in expanding its surface area and reaching the stationary configuration, whereas the other vacuole shrinks. In physiological conditions, such symmetry breaking can be triggered by either different nucleation conditions among the vacuoles or by shape fluctuations that can be due, among others, to natural inhomogeneities of the inverse bleb growth dynamics (these effects are, however, neglected in our model).

## Discussion

In summary, by identifying GV formation as inverse blebbing, we formulated a biophysical model of this process that can recapitulate all the characteristic morphological and dynamical features of GVs (i) to (iii). The model thus elucidates the mechanisms underlying the dynamics of GVs at coarse-grained level: Upon nucleation, both the GV and the cell adjust their configurations to reach mechanical equilibrium locally. Vacuole and cell surface tensions are modulated on their relative areal strains according to the following qualitative picture (Fig. 3A): For relative strains smaller than the threshold ${\varepsilon }^{\rm{*}}$ (corresponding to the size of the local membrane reservoir), the surface tension is in the cortex-dominated regime where all area increase is buffered by membrane reservoirs at constant tension. As shape changes are accommodated by active remodeling of the cell and bleb actomyosin cortices, tensional dynamic in this regime is governed by the characteristic timescale of actin turnover and myosin-II recruitment (18). Conversely, when these reservoirs are depleted, the surface tension enters the membrane-dominated regime where the area increase is buffered by the local mechanical stretch of the membrane. This mechanism induces an abrupt increase of the surface tension. Tension dynamic in this regime is governed by the characteristic timescale of membrane stretching, which is assumed to be a fast process. To all intents and purposes, we can consider it as instantaneous. For the case of a single GV, the picture simplifies. The cell, in fact, never enters the membrane-dominated regime (we verified this numerically up to pressures about 30 mmHg). Its surface tension is thus equal to ${\sigma }_0$ at all times during the lifetime of the GV.

Our results elucidate the multiscale complexity underlying the GV growth process: In response to externally applied macroscopic stresses (in the context of Schlemm’s canal, the hydrodynamic pressure), several microscopic, active, and passive, cellular processes are activated, including actin turnover, myosin-mediated contractile force generation, and unfolding of membrane reservoirs, which drive the mechanical shape change that the cell undergoes. In turn, these processes are self-regulated at the macroscale by the surface tension. In this respect, our results particularly highlight the role played by the membrane in determining the existence and stability of GVs at physiological pressures. Our analysis also show that the characteristic timescale induced by the viscous resistance exerted by the cell body on the inverse bleb is much larger than the characteristic timescale of actin turnover. While the latter is important to determine the growth characteristics at short times after nucleation, the former specifies the long-time behavior. In the context of glaucoma, our results challenge the established idea that cellular stiffness is the main determinant of GV formation, thus confirming previous phenomenological suggestions (10).

Our model further reveals that GVs are stabilized by the elastic response of the actomyosin cortex enveloping them upon reaching the steady state. This result indirectly specifies the possible pathways for GV collapse: GVs can shrink either if an apical pore is formed thus constituting, together with the meshwork pore, a transcellular channel through which aqueous humor can flow; or if an active contractile force is exerted on the vacuole by actin contractile structures additional to the cortical shell; or through coarsening (see below).

We remark that in our simulations we accounted for membrane reservoir sizes up to $100{\rm{\% }}$ as recently observed in experiments on doming 3D epithelia (17). However, these same experiments have discovered examples of endothelial cells with values of relative areal strain up to $300{\rm{\% }}$ (thus much larger than those currently accounted for in our work). These extreme deformations have been interpreted as manifestations of super-stretched cellular states. Our coarse-grained dynamical model holds formally also in this case, but the target tensions $\bar{\Sigma }$ and $\bar{\sigma }$ must account for the strain softening induced by cortical dilution at high strains underlying active super-elasticity (17). We neglected this mechanism here as we believe that it is irrelevant for GV formation.

We also remark that in our model, we assume the gap size parameter *a* (representing the pore mouth radius of GVs in vivo) to be a constant independent of the growth dynamics. This assumption precludes the model from capturing phenomena like the increase of the pore mouth diameter in time possibly due to the tearing of cellular adhesions; or its decrease possibly due to contractile forces exerted by F-actin polymerized locally around the pore. The latter pinching down of basal pores by F-actin could be especially important in the context of inverse bleb deflation to explain the formation of completely closed-off vacuoles (70, 71). We make this choice in the interest of simplicity because no clear-cut experimental evidence of such phenomena has been reported in the Literature so far. Nevertheless, we recall that our model predictions are robust against physiological variability of the model parameters, including *a* (Fig. 4B; see also SI Appendix Section V; Fig. S3). Therefore, we do not expect any qualitatively different results, even if the parameter *a* depends explicitly on the growth dynamics.

### Model approximations

The model defined by Eqs. (1), (3), and (4) relies on a few important approximations that we here recapitulate:

- The first approximation consists in assuming that the cell can add new focal adhesion complexes instantaneously during the perfusion process in order to cover its basal surface that expands in consequence of the inflation of inverse blebs.
- Another approximation is the assumption that inverse blebs have spherical cap shape. In fact, GVs in physiological conditions have ellipsoidal shape.
- To describe the overdamped dynamic of the inverse bleb radius, we have adopted the Rayleigh–Plesset equation without inertial terms. This is an approximated description. In fact, this equation was originally formulated to describe the dynamic of a vapor/gas bubble immersed in a fluid medium of infinite spatial extension. In our setting, instead, the inverse bleb grows in the fully confined space (i.e., confined in all spatial directions) of the cell inner body. This approximation is justified because the ratio of inverse bleb radius to cell radius is small, $( {r( t )/R( t )} ) \approx 0.1 - 0.2$. We can show that this condition is sufficient to make all additional correction terms in the Rayleigh–Plesset equation for the fully confined geometry negligible (72, 73) (SI Appendix Section VI).
- The coarse-grained description of the resistive forces exerted by the cell inner body through an effective viscous drag characterized by the dynamic viscosity parameter $\mu $ is also an approximation. To obtain a more refined description, one should model the forces exerted by each cellular component (like cytoskeletal elements and cellular organelles) explicitly.
- To define the steady-state value of the surface tension of an inverse bleb, we have defined the parameter *d* describing the typical size of the region in the basal aspect of the cell where the inverse bleb tension can equilibrate for times up to the typical lifetime of GVs. We have also assumed *d* to be constant in time. This is an approximated description of the real biological behavior as discussed in ref. (16). A more refined model should account explicitly for the diffusion of the inverse bleb tension. One way to implement this process is by considering *d* as a time-dependent variable that grow diffusively from the pore mouth radius, *a*. Alternatively, one can directly implement a diffusion equation for the inverse bleb tension, $\sigma $, equipped with suitable boundary conditions at the cell–inverse bleb interface. We leave the discussion of these refined models for future work.
- We have assumed the equilibration of the intracellular pressure, *P*, to be an instantaneous process, thus setting it equal to its equilibrium value (as specified by Laplace law) at each time. Likewise, we have also assumed the mechanical stretching of the cell membrane to be an instantaneous process, thus taking the limit ${\tau }_{\rm{m}} \to 0$. These are clearly approximations as real biological processes always have an associated timescale, no matter how small.
- To solve numerically the model equations, we typically assume the inverse bleb to be nucleated with a perfect hemispherical shape and with a surface tension, $\sigma \ = {\sigma }_{\rm{m}}\ $. This condition captures the experimental observation reported in ref. (44) that inverse blebbing is triggered by both the establishment of a pressure drop across the cell and a local weakening of the cell actin cortex. In our current model formulation, we make the approximation that the cortex is fully disrupted at nucleation. How the actual damage inferred to the cell actin cortex affects inverse bleb nucleation and/or growth is largely unknown. Intuitively, we can expect this aspect to be relevant to specify the fine details of the inverse bleb nucleation process, such as nucleation rates. These details are, however, not relevant for our model, because nucleation only enters our current description as a boundary condition. As regards growth, the model seemingly suggests that this parameter is not relevant, because the surface tension rapidly equilibrates to the initial surface tension, ${\sigma }_0$.

### Model predictions

The model provides the following novel predictions on GV dynamics:

- There exists a threshold pressure $\Delta {p}^*$ for GV formation. This pressure depends only on ${\varepsilon }^{\rm{*}}$ and on the cortical tension ${\sigma }_{\rm{c}}$ (through the cell initial surface tension ${\sigma }_0$) and is independent of GV geometry. In details, $\Delta {p}^{\rm{*}}$ decreases either if ${\sigma }_{\rm{c}}$ is reduced or if ${\varepsilon }^{\rm{*}}$ is increased. Conversely, if ${\sigma }_{\rm{c}}$ is increased or ${\varepsilon }^{\rm{*}}$ decreased, $\Delta {p}^{\rm{*}}$ increases. Moreover, an inverse relation between $\Delta {p}^{\rm{*}}$ and the vacuole sizes is shown: Decreasing $\Delta {p}^{\rm{*}}$ induces larger GVs in the membrane-dominated regime. These predictions suggest a few possible validating experiments: endothelial cells could be treated with myosin-impairing drugs (such as Blebbistatin) to reduce the contractility of the cortex and thus ${\sigma }_{\rm{c}}$; alternatively, lipids could be added ad hoc to increase the overall size of membrane reservoirs. In both cases, our model predicts increased GV sizes, a result that could be assessed via experimental measurements.
- At physiological pressure drops, there exists a threshold nucleation radius for GV growth of the order a few microns (about $1\ \mu {\rm{m}}$ at 7 mmHg).
- The timescale required for GV collapse, upon removal of the pressure drop, is larger than the corresponding timescale for GV growth at the same pressure conditions. Moreover, the model predicts that the characteristic timescale for GV collapse becomes longer if the cortical tension ${\sigma }_{\rm{c}}$ is reduced. Experimental evidence validating this prediction has been recently found (74).
- There exists a coarsening mechanism between multiple GVs inflating within the same cell, which is driven by the competition between these vacuoles for locally stretching the membrane. This competition generates a process similar to Ostwald ripening: For long times, symmetry breaking occurs and only one vacuole can grow toward the steady-state configuration at the expense of all the other ones that instead shrink. As this effect is strictly related to the interbleb competition for membrane, its experimental verification could be used as a proxy to test the role of the cell membrane during GV formation.

### Model limitations

We formulated here a coarse-grained biophysical model of GV formation as inverse blebbing. By its own nature, our model can only capture the macroscopic effects induced by the microscopic cellular processes involved in GV formation. This approximation captures the long-time morphological and dynamical features of GVs, but it fails to capture the characteristic processes occurring on short timescales. In this respect, our model does not contain any explicit elastic response contribution of the cell body on the inverse bleb (in fact, this term can also be shown to be negligible with respect to the effective viscous response). These short-time processes are relevant, particularly, to analyze the initial nucleation of GVs, which, at the level of the current discussion, only enters the theoretical description as a boundary condition. However, we believe that the current regime of focus is most relevant experimentally.

## Supplementary Material

pgac304_Supplemental_File

## Data Availability

All data presented in this study have been generated in silico using dedicated notebooks in Wolfram Mathematica. We have deposited all the codes and datasets in a public repository on GitHub—https://github.com/cairola/GiantVacuoleProject.git—to make them publicly available. We provide there also detailed instructions to use the codes. By using all this material, our results are fully reproducible.

## Footnotes

a In ref. (44), it is shown that inflating inverse blebs do not express any F-actin or myosin-II markers, thus demonstrating local cortex disruption at nucleation sites of inverse blebs.

b For inverse blebs with $0 < \theta < \pi /2$, the total volume is $\mathbin{\lower.3ex\hbox{$\buildrel<\over {\smash{\scriptstyle \sim}\vphantom{_x}}$}} 2\pi {a}^3/3 \simeq 0.03\ \mu {{\rm{m}}}^3$. This estimate is obtained by using the value of *a* reported in Table 1. This was measured in ref. (14) at the physiological pressure drop $\Delta p = 7\ {\rm{mmHg}}$. At these pressures, ${\rm{GV\ volume}} \simeq 15.67\ \mu {{\rm{m}}}^3$ according to the data from ref. (27). Given the much smaller size of these inverse blebs compared to empirically observed GVs, we neglect these solutions. A similar argument also holds at larger pressures. E.g., at $\Delta p = 14\ {\rm{mmHg}}$ Grierson and Lee measured $2a \simeq 0.9\ \mu {\rm{m}}$ (14), such that $( {2\pi {a}^3} )/3 \simeq 0.19\ \mu {m}^3$. At these pressures, the same Authors measured GVs with volume about $36.45\ \mu {{\rm{m}}}^3$.

c In ref. (16), the relaxation dynamic of membrane tension was studied by pulling tethers at fixed distance between one another and by monitoring their fluorescence, as a measure of surface tension, in response to a perturbation of one of the two tethers. These experiments demonstrate that in intact cells, for tethers as close as 5 μm, no correlation between the fluorescence signals are observed over up to 500 s. The two tethers are thus effectively independent at this timescale. Conversely, when measurements are repeated in cellular blebs, the signals are strongly correlated (lag time < 1 s). In this case, the tension almost instantaneously equilibrates in the tethers, in agreement with the fluid-mosaic model (55).

d Assuming cytoplasmic density $\rho \simeq 1{\rm{\ g/c}}{{\rm{m}}}^3$ (56) and viscosity $\mu \simeq 0.2 - 0.3\ {\rm{Pa}} \cdot {\rm{s}}$ (57), characteristic GV length $L \simeq 5 - 100\ \mu {\rm{m}}$ (19), and bleb growth rate $U = L/( {60{\rm{s}}} ) \simeq ( {0.08 - 2} )\ \mu {\rm{m/s}}$, we obtain the estimate for the Reynolds number ${\rm{Re}} = \rho UL/\mu \mathbin{\lower.3ex\hbox{$\buildrel\prec\over {\smash{\scriptstyle \sim}\vphantom{_x}}$}} {10}^{ - 6}$. Characteristic length and velocity of GVs are estimated according to the data in vitro from ref. (19). As these vacuoles are typically much larger than those observed in vivo (27), this calculation overestimates the Reynolds number. Therefore, we expect this assumption to hold also in physiological conditions.

e Larger cellular strains (up to 1,000%) have also been observed in the experiments of doming endothelial monolayers of ref. (17). However, cellular strains larger than about 100% are a manifestation of a super-elastic response (61), which we neglect in the present context.

f In this paper, by pulling membrane-attached beads with laser tweezers, the Authors show an exponential dependence of the surface tension of a membrane under mechanical stretching. The membrane enters this regime after having exhausted all its lipid reservoirs.

g To derive this relation, we set the relative area strain of the GV equal to the reservoir size ${\varepsilon }^{\rm{*}}$ and calculate from there *r* as a function of ${\varepsilon }^{\rm{*}}$. At the threshold, the steady-state surface tension is $\bar{\sigma } = {\sigma }_0$. The cell radius is obtained by volume conservation. The relation then follows from Eq. (2).
