# Supplementary material for: Model of inverse bleb growth explains giant vacuole dynamics during cell mechanoadaptation
Source: PNAS Nexus. 2022 Dec 23;2(2):pgac304. doi: 10.1093/pnasnexus/pgac304 (PMC9944300; doi:10.1093/pnasnexus/pgac304)
Supplement: pgac304_Supplemental_File [file pgac304_supplemental_file.docx]

**
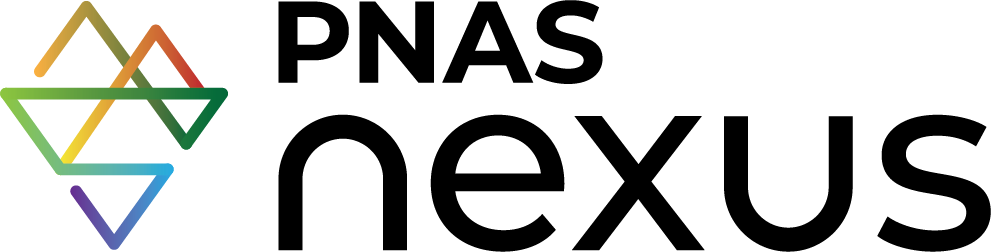
**

**Supplementary Information for**

Model of inverse bleb growth explains giant vacuole dynamics during cell mechanoadaptation

Andrea Cairoli, Alice Spenlehauer, Darryl R Overby and Chiu Fan Lee

Corresponding authors: Chiu Fan Lee and Andrea Cairoli

Email: [c.lee@imperial.ac.uk](mailto:c.lee@imperial.ac.uk) ; andrea.cairoli@crick.ac.uk

**This PDF file includes:**

Supplementary text

Figures S1 to S3

Table S1

SI References

**Supplementary Information Text**

**Introduction.** This *Supplementary Text* is organized as follows. Section I recapitulates the geometry of the perfused configuration and demonstrates how the vacuole pore contribution to the cell area and volume is negligible. In Section II, we characterize the mechanical equilibrium of the system after perfusion. We show that Laplace law holds for both the cell and the inverse bleb separately. The resulting equations, however, are not independent but are related through the intracellular pressure. We provide evidence that correction terms due to membrane bending are negligible for the giant vacuoles (GVs) typically observed *in vivo* in Schlemm's canal endothelial cells. In Section III, we study numerically the growth and collapse of GVs after reducing the cortical tension $\sigma_{c}$ (e.g., by treating the cell with the Rho-kinase Y-27632). We especially show that the timescale for GV collapse increases upon reducing the cortical tension compared to physiological conditions (main text). Section IV presents the linear stability analysis of the stationary configurations of giant vacuoles described by Eq. (2) (main text). In Section V, we study numerically how robust the growth process of GVs at physiological pressure drops is against physiological variability of the gap size parameter $a$. In Section VI, we calculate the asymptotic scaling of the rate of change of cell radius for sufficiently large vacuoles.

**I. Geometry of the perfused system**

The perfused system is defined as follows: (i) the vacuole is a spherical cap of radius $r$, opening angle $\theta$, satisfying the relation $r\sin\left( \theta\right)=a$, and symmetry axis equal to that of the cell body. (ii) The cell body is a sphere of radius $R$ (corrections due to the vacuole pore are negligible, see below). Vacuole surface area and volume are given by

| $S=2\pi r\left( 1-\cos\theta\right)$, $V=\frac{4}{3}\pi r^{3}\left( 2+\cos\theta\right)\left( \sin\frac{\theta}{2} \right)^{4},$ | (1) |
| --- | --- |

respectively. The total surface area of the cell is given by

| $A=4\pi R^{2}-\pi a^{2}$, | (2) |
| --- | --- |

including the contribution of the vacuole pore area, which must be subtracted from that of the cell. We now argue that this contribution can be conveniently neglected. We write

| $A=4\pi R^{2}\left[ 1-\frac{1}{4}\left( \frac{a}{R} \right)^{2} \right].$ | (3) |
| --- | --- |

However, $1-{a^{2}}/\left( 4R^{2} \right)\gtrsim1-0.002{R_{0}^{2}}/{R^{2}}\gtrsim0.998$ because ${R_{0}}/R\lesssim1$ in consequence of volume conservation during inverse bleb inflation. To obtain this estimate, we use typical values of the parameter $a$ at physiological pressures (main text, Table 1). Therefore, the area of the pore can be neglected. In consequence, we set the cell area to

| $A=4\pi R^{2}.$ | (4) |
| --- | --- |

A similar argument can be made also for the volume of the cell. Again, subtracting the contribution of the vacuole pore, this is given by

| $V=\frac{4\pi}{3}R^{3}-\frac{\pi h^{2}}{3}\left( 3R-h \right)$ | (5) |
| --- | --- |

with $h=R\left[ 1-\cos\left( \vartheta/2 \right) \right]$ and $\vartheta$ the angle subtending the pore diameter. However, because this angle is small, we can adopt the approximation, $\cos\left( \vartheta/2 \right)\approx1-{\vartheta^{2}}/8$, such that $h\approx R{\vartheta^{2}}/8\approx{R\left[ \sin\left( \vartheta/2 \right) \right]^{2}}/2\approx{a^{2}}/\left( 2R \right)$. Therefore,

| $V\approx\frac{4\pi}{3}R^{3}\left[ 1-\frac{1}{16}\left( \frac{a}{R} \right)^{4}\left( 3-\frac{a^{2}}{2R^{2}} \right) \right].$ | (6) |
| --- | --- |

However, the right-hand side of this equation is approximately one with an accuracy $\gtrsim{10}^{-5}$. Thus, we set the cell volume to

| $V=\frac{4\pi}{3}R^{3}.$ | (7) |
| --- | --- |

II. Mechanical equilibrium for the perfused system

In steady-state conditions, the mechanical equilibrium of the perfused system is specified as follows: (a) For the inflated cell, Laplace law holds. This relates the intracellular pressure to the cell radius as

| $P-P_{e}=\frac{2\sigma}{R}$ | (8) |
| --- | --- |

with $\sigma$ the surface tension of the cell (main text, Fig. 1). (b) For the inverse bleb, the corresponding mechanical equilibrium condition is determined by minimizing its free energy $F$ with respect to the independent variables $r$ and $\theta$. In complete generality, this free energy comprises (i) the surface energy of the inverse bleb $F_{S}$ (proportional to its area); (ii) the energy due to the perfusion pressure drop $F_{p}$ (proportional to its volume); (iii) the bending energy $F_{b}$ as specified by the Helfrich Hamiltonian [1]; and (iv) a Lagrange multiplier $L$ imposing the geometrical condition $r\sin\left( \theta\right)=a$. Putting those terms into equations, we write

| $\begin{matrix} F & = & F_{b}+F_{s}+F_{p}+L\left( r\sin\theta-a \right) \\ & = & 2\kappa_{m}\int H^{2}dA+\Sigma\int dA-\left( p-P \right)\int dV+L\left( r\sin\theta-a \right) \end{matrix}$ | (9) |
| --- | --- |

with $\kappa_{m}$ the membrane bending modulus, $H={-1}/r$ the mean curvature of the inverse bleb, $\Sigma$ its surface tension and $p-P$ the pressure drop at the cell-inverse bleb interface. Substituting area and volume of the inverse bleb, Eq. (1), we obtain

| $F=2\pi\left( 1-\cos\theta\right)\left\{ 2\kappa_{\text{m}}+r^{2}\left[ \Sigma-\frac{p-P}{3}r\left( 1-\cos\theta\left( 1+\cos\theta\right) \right) \right] \right\}+L\left( r\sin\theta-a \right).$ | (10) |
| --- | --- |

The equilibrium configuration is obtained by solving $\partial_{r}F=0=\partial_{\theta}F$. In details,

| $\frac{\partial F}{\partial r}=2\pi r\left( 1-\cos\theta\right)\left[ 2\Sigma-\left( p-P \right)r\left( 1+\frac{1}{2}\cos\theta\left( 1+\cos\theta\right) \right) \right]+L \sin\theta,$ | (11) |
| --- | --- |
| $\frac{\partial F}{\partial\theta}=4\pi\kappa_{\text{m}}\sin\theta+2\pi r^{2}\sin\theta\left[ \Sigma-\frac{\left( p-P \right)r}{2}{(sin \theta)}^{2} \right]+Lr\cos\theta.$ | (12) |

Setting these equations to zero yields the equilibrium condition

| $p-P=\frac{2\Sigma}{r}+\frac{4\kappa_{\text{m}}}{r^{3}}\frac{1+\cos\theta}{1-\cos\theta}.$ | (13) |
| --- | --- |

The free energy of the inverse bleb is thus obtained by substituting Eq. (13) into Eq. (10). We then obtain

| $F=\frac{2\pi}{3}\Sigma r^{2}-\frac{2\pi}{3}\Sigma r^{2}\left( 1-\frac{a^{2}}{r^{2}} \right)\cos\theta+4\pi\kappa_{\text{m}}\left( 1-\frac{2}{3}\frac{a^{2}}{r^{2}} \right)-4\pi\kappa_{\text{m}}\left( 1+\frac{a^{2}}{3r^{2}} \right)\cos\theta.$ | (14) |
| --- | --- |

Inverse bleb configurations exist for all $r$ because $F\geq0$ (recall that $\theta\in\left. \left[ \pi/2,\pi\right. \right)$).

We now show that the bending term in the rhs of Eq. (13) can be effectively neglected. The contribution due to bending is expected to be negligible for sufficiently large $r$, such that we can use the expansion $\cos\theta\simeq1-{a^{2}}/\left( 2r^{2} \right)$. Substituting this expression into Eq. (13), we can write

| $p-P=\frac{2\Sigma}{r}+\frac{4\kappa_{\text{m}}}{r^{3}}\frac{1-\left\vert\cos\theta\right\vert}{1+\left\vert\cos\theta\right\vert}\simeq\frac{2\Sigma}{r}\left( 1+\frac{\kappa_{\text{m}}}{2\Sigma}\frac{a^{2}}{r^{4}} \right),$ | (15) |
| --- | --- |

where the second term in parentheses satisfies $\kappa_{m}{a^{2}}/\left( 2\Sigma r^{4} \right)≾{\kappa_{m}}/\left( 2\sigma_{0}r^{2} \right)$. This term can thus be neglected when ${\kappa_{m}}/\left( 2\sigma_{0}r^{2} \right)≾1$, which yields $r≿\sqrt{{\kappa_{m}}/\left( 2\sigma_{0} \right)}$. For typical values of $\kappa_{m}$ and $\sigma_{0}$ ($\sigma_{0}\simeq414 \text{pN/}\mu\text{m}$ [2]; $\kappa_{\text{M}}\simeq43 \text{pN}\cdot\text{nm}$ [3]), this yields $r≿0.007\mu\text{m}$, which is satisfied for the inverse blebs considered here (in fact $r\geq a$).

Therefore, the bending term in Eq. (13) can be neglected. The mechanical equilibrium of the inverse bleb is thus described by the Laplace law

| $p-P=\frac{2\Sigma}{r}.$ | (16) |
| --- | --- |

III. Growth and collapse of giant vacuoles upon cortical tension impairment

In Fig. S1 we study numerically how the growth and collapse processes of GVs are affected by a reduction of the cortical tension $\sigma_{c}$. This can be realized experimentally, e.g., by treating Schlemm’s canal endothelial cells with the Rho-kinase inhibitor Y-27632, which induces cell relaxation and disassembly of actin stress fibers and focal adhesions (thus reducing $\sigma_{c}$) [4] [5] [6] by inhibiting the phosphorylation of the regulatory myosin light chain [7] [8] [9] [10]. Numerical solutions of model equations reveal: a slight decrease of the characteristic timescale for GV inflation (about $1.2-1.6 \min$ in this case versus about $1.6-2.2 \min$ for the wildtype); and a significant increase of the characteristic timescale for GV collapse upon removal of the pressure drop (about $4-6 \min$ for the wildtype versus about $8-10 \min$ for the treated case).

IV. Linear stability analysis of the stationary configurations of giant vacuoles

The linear stability of the solutions of Eq. (2) (main text) is studied by computing the radial force

| $f=p-P-\frac{2\Sigma}{r} ,$ | (17) |
| --- | --- |

which is exerted on the surface of the GV in response to a small positive perturbation of its radius. Positive force indicates an unstable configuration; vice versa, negative force indicates a stable configuration.

We call $\left( r^{\text{*}},\theta^{\text{*}},R^{\text{*}},P^{\text{*}},\bar{\sigma}\text{*} \right)$ the stationary configuration of the perfused system obtained by solving Eq. (2) (main text) complemented with volume conservation and a suitable functional prescription for $\bar{\Sigma}$ and $\bar{\sigma}$ (main text, Fig. 3 A). We now consider a small positive perturbation $\delta r$ of the vacuole radius and denote the perturbed solutions accordingly as $r=r^{\text{*}}+\delta r$, $\theta=\theta^{\text{*}}+\delta\theta$, $R=R^{\text{*}}+\delta R$, $P-P_{e}=P^{\text{*}}+\delta P$, $\sigma=\bar{\sigma}\text{*}+\delta\sigma$ and $\Sigma=\bar{\Sigma}\text{*}+\delta\Sigma$. Vacuole and cell perturbed surface tension are given by

| $\Sigma=\bar{\Sigma}+2E_{\text{C}}h_{\text{C}}\left( 1-\frac{r^{\text{*}}}{r} \right), \sigma=\bar{\sigma},$ | (18) |
| --- | --- |

where for the inverse bleb we also consider the elastic resistance to deformations due to additional contractile actomyosin structures (here assumed to envelop the bleb and of thickness $h_{C}$). The angular perturbation follows from the geometrical constraint, $r\sin\theta=a$, i.e.,

| $\theta=\pi-\sin^{-1} \left( \frac{a}{r^{\text{*}}+\delta r} \right)\simeq\pi-\sin^{-1} \left[ \frac{a}{r^{\text{*}}}\left( 1-\frac{\delta r}{r^{\text{*}}} \right) \right]\simeq\theta^{\text{*}}+\frac{\sin\theta^{\text{*}}}{\left\vert\cos\theta^{\text{*}} \right\vert}\frac{\delta r}{r^{\text{*}}}.$ | (19) |
| --- | --- |

Using this expansion, we find

| $\cos\theta\simeq\text{cos}\left( \theta^{\text{*}}+\frac{\sin\theta^{\text{*}}}{\left\vert\cos\theta^{\text{*}} \right\vert}\frac{\delta r}{r^{\text{*}}} \right)\simeq\cos\theta^{\text{*}}-\frac{\left( \sin\theta^{\text{*}} \right)^{2}}{\left\vert\cos\theta^{\text{*}} \right\vert}\frac{\delta r}{r^{\text{*}}}.$ | (20) |
| --- | --- |

Volume conservation yields the cell radius perturbation. Expanding the equation $R^{3}=R_{0}^{3}+2r^{3}\left( 2+\cos\theta\right)\text{sin}^{4}\left( \theta/2 \right)$ to first order in $\delta r$ and $\delta R$, we obtain

| $\delta R=\frac{r^{\text{*}2}}{4R^{\text{*}2}}\left( 1-\cos\theta^{\text{*}} \right)^{2}\left[ 2+\cos\theta^{\text{*}}+2\frac{\left( 1+\cos\theta^{\text{*}} \right)^{2}}{\left\vert\cos\theta^{\text{*}} \right\vert} \right]\delta r.$ | (21) |
| --- | --- |

We note that this quantity is positive for all $\theta^{*}$. Similarly expanding Eqs. (1), we find the perturbation of the surface tensions, i.e.,

| $\delta\Sigma={\bar{\Sigma}\text{'}}^{*}\delta S+\frac{2E_{C}h_{C}}{r^{*}}\delta r, \delta\sigma={\bar{\sigma}\text{'}}^{*}\delta A,$ | (22) |
| --- | --- |

with ${\bar{\Sigma}\text{'}}^{*}=\partial_{S}\bar{\Sigma}\left( S^{*} \right)$ and $\delta S$ the perturbation of its area; likewise, ${\bar{\sigma}\text{'}}^{*}=\partial_{A}\bar{\sigma}\left( A^{*} \right)$ and $\delta A$ the perturbation of its area. Area perturbations of inverse bleb and cell are given by

| $\delta S=2\pi\left[ 2\left( 1-\cos\theta^{\text{*}} \right)+\frac{\left( \sin\theta^{\text{*}} \right)^{2}}{\left\vert\cos\theta^{\text{*}} \right\vert} \right]r^{\text{*}}\delta r, \delta A=6\pi R^{\text{*}}\delta R,$ | (23) |
| --- | --- |

also positive for all $\theta^{*}$. For the intracellular pressure we expand the Laplace law $P-P_{e}={2\sigma}/R$, which yields

| $\delta P=\frac{2}{R^{\text{*}}}\delta\sigma-\frac{2\bar{\sigma}\text{*}}{R^{\text{*}2}}\delta R.$ | (24) |
| --- | --- |

The radial force exerted on the GV surface is thus given by

| $f\simeq-\delta P-\frac{2}{r^{\text{*}}}\delta\Sigma+\frac{2\bar{\sigma}\text{*}}{r^{\text{*}2}}\delta r$ | (25) |
| --- | --- |

where we employed the equilibrium relation $p-P^{\text{*}}-2\bar{\Sigma}\text{*}/{r^{\text{*}}}=0$. Combining Eqs. (23), (24) and (25) finally yields

| $\frac{f}{\delta r}=\frac{2\left( \bar{\Sigma}\text{*}-2E_{\text{C}}h_{\text{C}} \right)}{r^{\text{*}2}}-\frac{2{\bar{\Sigma}\text{'}}^{*}}{r^{\text{*}}}\frac{\delta S}{\delta r}-\frac{2{\bar{\sigma}\text{'}}^{*}}{R^{\text{*}}}\frac{\delta A}{\delta r}-\frac{2\bar{\sigma}\text{*}}{R^{\text{*}2}}\frac{\delta R}{\delta r}$ | (26) |
| --- | --- |

We consider first the case when there are no additional contractile structures on the inverse blebs, i.e., $h_{C}=0$. For configurations of inverse blebs in the cortex-dominated regime, the steady-state surface tensions of the inverse blebs and cell are assumed constant, $\bar{\Sigma}\text{*}=\bar{\sigma}\text{*}=\sigma_{0}$. As such, ${\bar{\Sigma}\text{'}}^{*}={\bar{\sigma}\text{'}}^{*}=0$. Eq. (26) thus reduces to

| $\frac{f}{\delta r}=\frac{2\sigma_{0}}{r^{\text{*}2}}\left( 1-\frac{r^{\text{*}2}}{R^{\text{*}2}}\frac{\delta R}{\delta r} \right)$ | (27) |
| --- | --- |

This quantity is positive for all $r^{*}$; therefore, these inverse bleb configurations are unstable. By thinking in terms of dynamical systems theory, we expect configurations of inverse blebs in the membrane-dominated regime to be stable. However, there is no similar simple analytical argument to demonstrate this. We can then study numerically Eq. (26). Fig. S2A confirms that vacuoles in the cortex-dominated regime are unstable (force is positive); Fig. S2B indeed shows that vacuoles in the membrane-dominated regime are stable as expected (force is negative).

Eq. (26) also enables us to estimate the thickness of additional cortex that the cells need to build to stabilize vacuoles in the cortex-dominated regime. Imposing the force to be negative, and solving with respect to $h_{C}$ we find

| $h_{\text{C}}\geq\frac{\sigma_{0}}{2E_{\text{C}}}\left[ 1-\left( \frac{r^{\text{*}}}{R^{\text{*}}} \right)^{2}\frac{\delta R}{\delta r} \right]$ | (28) |
| --- | --- |

Recalling that ${\delta R}/{\delta r\propto\left( {r^{\text{*}}}/{R^{\text{*}}} \right)^{2}}$, the term in brackets is circa one. Therefore, we obtain the approximate expression $h_{\text{C}}\gtrsim{\sigma_{0}}/{2E_{\text{C}}}\approx23 n\text{m}$.

V. Robustness of the growth dynamics of giant vacuoles against physiological variability of the gap size parameter at physiological pressure drops

In our model we assume the gap size parameter $a$ (representing the pore mouth diameter of GVs in vivo) to be a constant independent of the growth dynamics. This assumption precludes the model from capturing phenomena like the increase of the pore mouth diameter in time possibly due to tearing of cellular adhesions; or its decrease possibly due to contractile forces exerted by F-actin polymerized locally around the pore. Nevertheless, we recall that our model predictions are robust against physiological variability of the model parameters, including $a$ (main text, Fig. 4 B). These results support the idea that, even if $a$ changed over time during growth, we would expect to observe the same qualitative dynamical behaviour as that shown in Fig. 3 C (main text), but with target steady-state configurations of different sizes and adapting over time as a function of $a$. Correspondingly, also the timescale to reach the steady-state configuration would become time-dependent and a function of $a$. To further support this result, we recall that in our model equations the parameter $a$ only enters the equation for the inverse bleb surface area. We rewrite this as follows:

| $S=2\pi r^{2}\left( 1-\cos\theta\right)+\pi d^{2}\left[ 1-\left( \frac{a}{d} \right)^{2} \right].$ | (29) |
| --- | --- |

Using the measurements of maximum meshwork pore width in fixated eyes at large pressure drops (30 mmHg) reported in ref. [11], and the minimum value for the parameter $d$ (Table 1, main text), we obtain $\mathbf{1}\boldsymbol{-}\left( \boldsymbol{a}/\boldsymbol{d} \right)^{\boldsymbol{2}}\boldsymbol{\approx0.97}$. The correction term is thus very small, even under conditions of extremely elevated (not physiological) pressure drops. We also confirm this result numerically in Fig. S3. Note that for each simulation therein, $a$ is kept constant, i.e., we are not implementing a time dependent dynamic for the parameter.

VI. Effects of confined geometry on the dynamic of inverse bleb radius

The Rayleigh-Plesset equation was originally derived to describe the growth of a spherical bubble immersed in a fluid medium with infinite spatial extension. This setting is different than that observed during inverse blebbing, where the inverse bleb (the counterpart of the bubble just mentioned) grows in the confined space of the cell inner body. To understand the effects of the confined geometry of the cell on inverse bleb growth, we consider the similar setting where a spherical bubble grows centred in a spherical fluid drop. This problem has been characterized in refs. [12] [13]. The dynamic of the bubble radius was described there in terms of an extended Rayleigh-Plesset equation in the presence of both viscous dissipative forces and tension forces at the bubble-liquid interface. Written in our own notation and neglecting inertial terms (main text), the equation reads as below:

| $4\nu\left[ \frac{1}{r(t)}\frac{dr(t)}{dt}-\frac{1}{R(t)}\frac{dR(t)}{dt} \right]=\left[ 1-\frac{r(t)}{R(t)} \right]^{3}\left[ p-P\left( t \right)-\frac{2\Sigma\left( t \right)}{r\left( t \right)} \right] .$ | (30) |
| --- | --- |

This equation differs from the Rayleigh-Plesset equation in unconfined medium in two main aspects: First, the numerical prefactor contains a multiplicative correction that is function of the ratio $\left( {r(t)}/{R(t)} \right)$. Second, the equation contains an additional term accounting for the rate of change of the cell radius.

We can estimate the rate of change of the cell radius exactly in our setting by taking the time derivative of the volume conservation relation $R^{3}=R_{0}^{3}+2r^{3}\left( 2+\cos\theta\right){\sin\left( \theta/2 \right)}^{4}$. This yields the following equation:

| $\frac{dR}{dt}=2\left( \frac{r}{R} \right)^{2}\left\{ \left( 2+\cos\theta\right)\left( \sin\left( \frac{\theta}{2} \right) \right)^{4}\frac{dr}{dt}+\frac{r}{3}\left( \sin\left( \frac{\theta}{2} \right) \right)^{3}\left[ 2\cos\left( \frac{\theta}{2} \right)+\cos\left( \frac{3}{2}\theta\right) \right]\frac{d\theta}{dt} \right\},$ | (31) |
| --- | --- |

with the angular derivative defined as

| $\frac{d\theta}{dt}=\frac{-1}{\sqrt{1-\left( \frac{a}{r} \right)^{2}}}\frac{a}{r^{2}}\frac{dr}{dt}.$ | (32) |
| --- | --- |

Putting both equations together, we obtain

| $\frac{dR}{dt}=2\left( \frac{r}{R} \right)^{2}\left( \sin\left( \frac{\theta}{2} \right) \right)^{3}\left\{ \left( 2+\cos\theta\right)\sin\left( \frac{\theta}{2} \right)-\frac{a}{3r}\left( 1-\left( \frac{a}{r} \right)^{2} \right)^{-1/2}\left[ 2\cos\left( \frac{\theta}{2} \right)+\cos\left( \frac{3\theta}{2} \right) \right] \right\}\frac{dr}{dt}.$ | (33) |
| --- | --- |

For large vacuoles, $r\gg a$, the equation reduces to

| $\frac{dR}{dt}=2\left( \sin\left( \frac{\theta}{2} \right) \right)^{4}\left( 2+\cos\theta\right)\left( \frac{r}{R} \right)^{2}\frac{dr}{dt}.$ | (34) |
| --- | --- |

This equation is of the form ${dR(t)}/{dt}=\Phi\left( \theta\right)\left( {r(t)}/{R(t)} \right)^{2}{dr(t)}/{dt}$ with a numerical prefactor $0\leq\Phi\left( \theta\right)\leq6$.

Typical GVs in physiological conditions have volumes about $15.67 {\mu m}^{3}$ according to the data from ref. [14]. This corresponds to radii about $1.55 \mu m$. At the same time, Schlemm’s canal cells have typical radii about $9-11 \mu m$ (main text). These values yield $\left( {r(t)}/{R(t)} \right)\approx0.1-0.2$. Therefore, all corrections due to the confined geometry of our setting can be neglected.


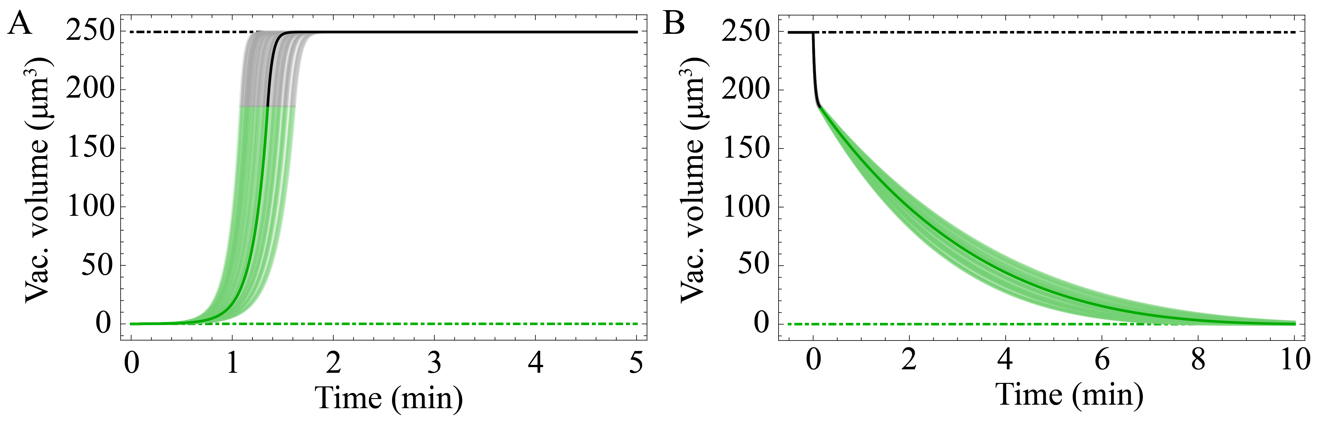


**Fig. S1.** Growth and collapse of giant vacuoles upon reduction of the cortical tension. We set $\boldsymbol{\sigma}_{\text{c}}\boldsymbol{=}187 \text{pN/}\mu\text{m}$ (half of value in physiological conditions). Pressure protocols, initial conditions and other model parameters are the same as in Fig. 3 C (main text). (A) Giant vacuole growth. The characteristic timescale for vacuole inflation is decreased compared to wildtype (about $1.6-2.2 \min$). (B) Giant vacuole collapse. The characteristic timescale for vacuole deflation is significantly increased compared to wildtype (about $4-6 \min$).

**
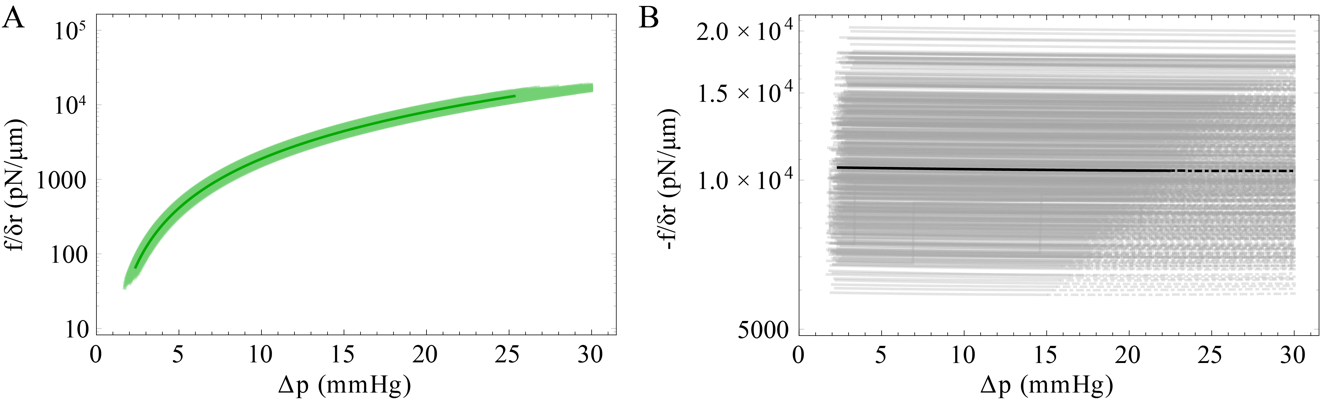
**

**Fig. S2. Linear stability of giant vacuoles.** The linear stability of a giant vacuole against small positive perturbations of its radius is investigated by calculating numerically the response force exerted on the vacuole, Eq. (17). Reference curves (darker lines) correspond to the choice of model parameters (same as in Fig. 4 A, main text): $R_{0}=10 \mu\text{m}$, $a=0.25 \mu\text{m}$, $d=10 \mu\text{m}$, $\sigma_{\text{m}}=40 \mathrm{pN}/\mu\text{m}$, $\sigma_{\text{c}}=374 \text{pN}/\mu\text{m}$, $\varepsilon^{*}=0.5$ and $K_{\text{m}}={10}^{5} \text{N}/\text{m}$. Lytic values of the surface tension (dot-dashed line) corresponds to a local relative areal strain $5\text{\%}\text{ }\varepsilon^{\text{*}}$. Blurred regions correspond to values obtained by allowing for up to $20\%$ variability in the parameters (according to Table 1, main text). A. Inverse blebs in the cortex-dominated regimes. B. Inverse blebs in the membrane-dominated regime.

**
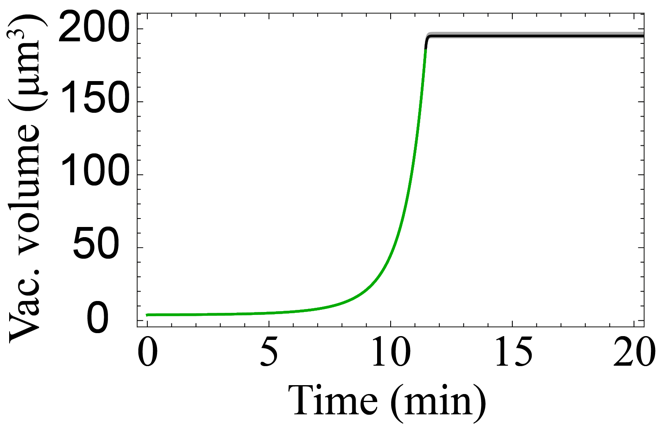
**

**Fig. S3. Robustness of the growth dynamics of giant vacuoles against physiological variability of the gap size parameter at physiological pressure drops.** Pressure protocols, initial conditions and other model parameters are the same as in Fig. 3 C (main text). Blurred regions correspond to trajectories obtained by allowing for up to $95\%$ variability in the gap size parameter, $a$. We plot simulation results for 100 different values of the parameter chosen randomly.

**Table S1. Summary of the model notation.**

| Parameter | Description | Units |
| --- | --- | --- |
| $\Delta p$ | Perfusion pressure drop | $\text{mmHg}$ |
| $P_{e}$ | Ambient pressure | $\text{mmHg}$ |
| $R_{0}$ | Cell radius (initial configuration) | $\mu\text{m}$ |
| $2a$ | Pore diameter | $\mu\text{m}$ |
| $\sigma_{m}$ | Cell membrane in-plane tension | $\text{pN/}\mu\text{m}$ |
| $K_{m}$ | Cell membrane area expansion modulus | $\text{N/m}$ |
| $\sigma_{c}$ | Cell cortex in-plane tension | $\text{pN/}\mu\text{m}$ |
| $\sigma_{0}$ | Cell surface tension (initial configuration) | $\text{pN/}\mu\text{m}$ |
| $P_{0}$ | Intracellular pressure (initial configuration) | $\text{mmHg}$ |
| $p$ | Pressure inside the filter | $\text{mmHg}$ |
| $r$ | Inverse bleb radius | $\mu\text{m}$ |
| $\theta$ | Inverse bleb opening angle | $\text{adim}$ |
| $\Sigma$ | Inverse bleb surface tension | $\text{pN/}\mu\text{m}$ |
| $R$ | Cell radius (perfused configuration) | $\mu\text{m}$ |
| $P$ | Intracellular pressure (perfused configuration) | $\text{mmHg}$ |
| $\sigma$ | Cell surface tension (perfused configuration) | $\text{pN/}\mu\text{m}$ |
| $d$ | Radius of tension equilibrating membrane patch | $\mu\text{m}$ |
| $\mu$ | Effective dynamic viscosity of inner cell body | $\text{pN}\text{∙s}\text{/}{\mu\text{m}}^{2}$ |
| $\bar{\Sigma}$ | Inverse bleb surface tension (steady-state value) | $\text{pN/}\mu\text{m}$ |
| $\bar{\sigma}$ | Cell surface tension (steady-state value) | $\text{pN/}\mu\text{m}$ |
| $\varepsilon_{C}$ | Relative cell area strain | $\text{adim}$ |
| $A$ | Cell surface area (perfused configuration) | $\mu\text{m}^{2}$ |
| $A_{0}$ | Cell surface area (initial configuration) | $\mu\text{m}^{2}$ |
| $\varepsilon_{B}$ | Relative inverse bleb area strain | $\text{adim}$ |
| $S$ | Inverse bleb surface area (perfused configuration) | $\mu\text{m}^{2}$ |
| $S_{0}$ | Inverse bleb surface area (initial configuration) | $\mu\text{m}^{2}$ |
| $\varepsilon^{*}$ | Maximum relative area strain buffered by membrane reservoirs | $\text{adim}$ |
| $\tau_{c}$ | Characteristic timescale of actin turnover | $\text{s}$ |
| $\tau_{m}$ | Characteristic timescale of tension equilibration in a taut membrane | $\text{s}$ |
| $S^{*}$ | Maximum relative inverse bleb area strain buffered by membrane reservoirs | $\mu\text{m}^{2}$ |
| $\Delta p^{\text{*}}$ | Giant vacuole nucleation pressure | $\text{mmHg}$ |
| $\Delta p^{\dagger}$ | Minimum pressure drop for vacuole growth independent of nucleation radius | $\text{mmHg}$ |

**SI References**

| [1] | W. Helfrich, “Elastic properties of lipid bilayers: theory and possible experiments.,” *Z Naturforsch C,* vol. 28, pp. 693-703, 1973. |
| --- | --- |
| [2] | J. Y. Tinevez, U. Schulze, G. Salbreux, J. Roensch, J. F. Joanny and E. Paluch, “Role of cortical tension in bleb growth.,” *Proc Natl Acad Sci,* vol. 106, no. 44, pp. 18581-18586, 2009. |
| [3] | R. Phillips, J. Kondev, J. Theriot and H. Garcia , Physical biology of the cell, New York: Garland Science, 2012. |
| [4] | P. R. Vasantha, P. F. Deng, J. Kumar and D. L. Epstein, “Modulation of aqueous humor outflow facility by the rho kinase–specific inhibitor Y-27632,” *Invest Ophthalmol Vis Sci,* vol. 42, no. 5, pp. 1029-1037, 2001. |
| [5] | M. Honjo, H. Tanihara, M. Inatani, N. Kido, T. Sawamura, B. Y. Yue, S. Narumiya and Y. Honda, “Effects of rho-associated protein kinase inhibitor Y-27632 on intraocular pressure and outflow facility,” *Invest Ophthalmol Vis Sci,* vol. 42, no. 1, pp. 137-144, 2001. |
| [6] | R. Rosenthal, L. Choritz, S. Schlott, N. E. Bechrakis, J. Jaroszewski, M. Wiederholt and M. Thieme, “Effects of ML-7 and Y-27632 on carbachol-and endothelin-1-induced contraction of bovine trabecular meshwork,” *Exp Eye Res,* vol. 80, no. 6, pp. 837-845, 2005. |
| [7] | S. P. Davies, H. Reddy , M. Caivano and P. Cohen, “Specificity and mechanism of action of some commonly used protein kinase inhibitors.,” *Biochem J,* vol. 351, no. 1, pp. 95-105, 2000. |
| [8] | T. Ishizaki, M. Uehata, I. Tamechika, J. Keel, K. Nonomura, M. Maekawa and S. Narumiya, “Pharmacological properties of Y-27632, a specific inhibitor of rho-associated kinases,” *Mol Pharmacol,* vol. 57, no. 5, pp. 976-983, 2000. |
| [9] | M. Uehata, T. Ishizaki, H. Satoh, T. Ono, T. Kawahara, T. Morishita, H. Tamakawa, K. Yamagami, J. Inui , M. Maekawa and et al, “Calcium sensitization of smooth muscle mediated by a rho-associated protein kinase in hypertension,” *Nature,* vol. 389, no. 6654, p. 990, 1997. |
| [10] | K. Kaibuchi, S. Kuroda and M. Amano, “Regulation of the cytoskeleton and cell adhesion by the rho family gtpases in mammalian cells,” *Annu Rev Biochem,* vol. 68, no. 1, pp. 459-486, 1999. |
| [11] | I. Grierson and W. R. Lee, “Pressure effects on flow channels in the lining endothelium of Schlemm’s canal. A quantitative study by transmission electron microscopy.,” *Acta Ophthalmol,* vol. 56, pp. 935-952, 1978. |
| [12] | O. Vincent and P. Marmottant, “On the statics and dynamics of fully confined bubbles,” *J Fluid Mech,* vol. 827, pp. 194-224, 2017. |
| [13] | K. Leonov and I. Akhatov, “Towards a theory of dynamics of a single cavitation bubble in a rigid micro-confinement,” *Int J Multiph Flow,* vol. 130, p. 103369, 2020. |
| [14] | I. Grierson and W. R. Lee, “Light microscopic quantitation of the endothelial vacuoles in Schlemm’s canal.,” *Am J Ophthalmol,* vol. 84, no. 2, pp. 234-246, 1977. |
